# Exosomes as Rheumatoid Arthritis Diagnostic Biomarkers and Therapeutic Agents

**DOI:** 10.3390/vaccines11030687

**Published:** 2023-03-17

**Authors:** Romina Heydari, Fatemeh Koohi, Milad Rasouli, Kimia Rezaei, Elham Abbasgholinejad, Sander Bekeschus, Mohammad Doroudian

**Affiliations:** 1 Department of Cell and Molecular Sciences, Faculty of Biological Sciences, Kharazmi University, Tehran 14911-15719, Iran; 2 Endocrinology and Metabolism Research Center, Endocrinology and Metabolism Clinical Sciences Institute, Tehran University of Medical Sciences, Jalale-Al-Ahmad Ave, 1411713137 Tehran, Iran; 3 Department of Physics, Kharazmi University, 49 Dr. Mofatteh Ave, Tehran 15614, Iran; 4 ZIK Plasmatis, Leibniz Institute for Plasma Science and Technology (INP), Felix-Hausdorff-Str 2, 17489 Greifswald, Germany; sander.bekeschus@inp-greifswald.de

**Keywords:** autoimmunity, extracellular vesicles, EVs, mesenchymal stem cells

## Abstract

Rheumatoid arthritis (RA) is a chronic inflammatory joint disorder that causes systemic inflammation, autoimmunity, and joint abnormalities that result in permanent disability. Exosomes are nanosized extracellular particles found in mammals (40–100 nm). They are a transporter of lipids, proteins, and genetic material involved in mammalian cell–cell signaling, biological processes, and cell signaling. Exosomes have been identified as playing a role in rheumatoid arthritis-related joint inflammation (RA). Uniquely functioning extracellular vesicles (EVs) are responsible for the transport of autoantigens and mediators between distant cells. In addition, paracrine factors, such as exosomes, modulate the immunomodulatory function of mesenchymal stem cells (MSCs). In addition to transporting genetic information, exosomes convey miRNAs between cells and have been studied as drug delivery vehicles. In animal models, it has been observed that MSCs secrete EVs with immunomodulatory properties, and promising results have been observed in this area. By understanding the diversity of exosomal contents and their corresponding targets, it may be possible to diagnose autoimmune diseases. Exosomes can be employed as diagnostic biomarkers for immunological disorders. We here discuss the most recent findings regarding the diagnostic, prognostic, and therapeutic potential of these nanoparticles in rheumatoid arthritis and provide an overview of the evidence pertaining to the biology of exosomes in RA.

## 1. Introduction

Rheumatoid arthritis (RA) is a joint-affecting inflammatory disease. It is characterized by joint deterioration, inflammation, and an increasing number of other health complications. In industrialized nations, 5–50 new cases of RA are detected per 100,000 people per year by inflammation, joint degeneration, and increasing comorbidity. According to epidemiological data, RA affects 0.5–1% of adults and worsens their quality of life. RA is more prevalent among women and the elderly; nevertheless, people of all ages can be impacted by this disease [1,2]. In addition to genetic and familial variables, many environmental, nutritional, and lifestyle factors have been proven to raise the risk of RA. Interleukin (IL)-6, interleukin IL-1β, and tumor necrosis factor (TNF)-α have been linked to the development of RA [3,4]. The interaction between macrophages, synoviocytes, and osteoclasts is responsible for joint inflammation and peripheral bone destruction [5]. Pluripotent cytokines such as interleukin-6 (IL-6) are important pro-inflammatory mediators. IL-6 is essential for immune activation and inflammation in RA. Therefore, inhibiting IL-6 is an attractive method for controlling RA [6]. Most modern RA therapies limit inflammation, reduce joint degeneration, and prevent bone loss. Nonspecific treatments for RA include nonsteroidal anti-inflammatory drugs (NSAIDs), glucocorticoids, and disease-modifying antirheumatic medications (DMARDs). Due to their nonselective activity, they may cause undesirable consequences other than the immunological response over the long term [7].

**Figure 1:**
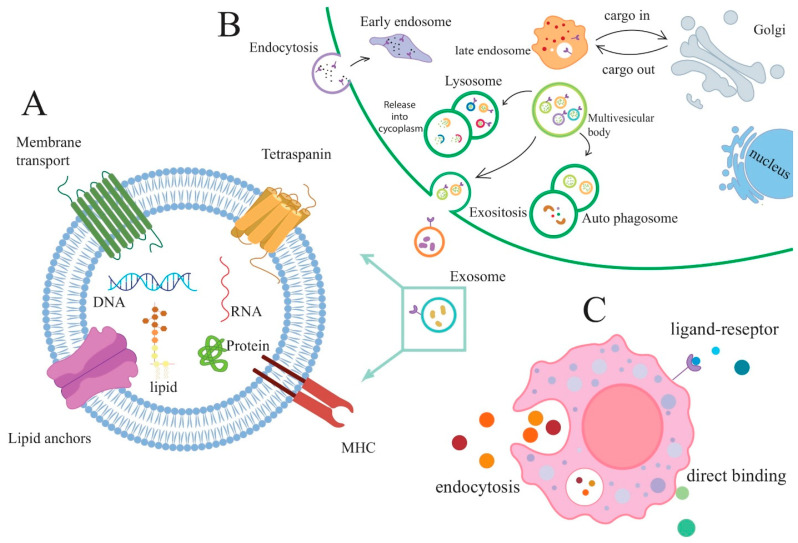
(**A**) Exosomes package their cargo in intraluminal vesicles (ILVs), which contain RNA, lipids, proteins, and receptors. (**B**) The biogenesis of exosomes starts with endosomal configuration. By depression and endocytosis of the plasma membrane, extracellular material enters the cytoplasm, fuses with early endosomes, the endoplasmic reticulum, and performed Golgi bodies, and produces late endosomes, or MVBs. Two different fates will be assigned to the generated MVBs. MVB contains intraluminal vesicles that are degraded by the lysosome in some instances. As an alternative, MVBs, called exosomes, travel to the cell’s surface and evacuate intraluminal vesicles of their content. (**C**) An exosome can attach to receptors on the surface of target cells, bind to endocytosis, or directly bind to recipient cells.

Extracellular vesicles (exosomes) with sizes between 40 and 100 nm have recently been discovered to be secreted by mammalian cells. Multiple lines of evidence suggest that extracellular vesicles facilitate cell–cell communication [8,9,10]. Numerous advantages exist between exosomes and synthesized nanoparticles. Compared to conventional liposomes and manufactured nanoparticles, exosomes are more biocompatible and readily absorbed by target cells due to their membrane proteins and endogenous origin. Exosomes are more stable in bodily fluids than similarly structured liposomes. For instance, macrophages and reticuloendothelial cells can directly or indirectly eliminate liposomes. Moreover, multiple investigations have demonstrated that exosomes avoid the immune system and prolong circulation time [11,12,13]. Nucleic acids (DNA, ribonucleic acid, micro-RNAs, and long noncoding RNAs (lncRNAs)), lipids, metabolites, proteins, cytosolic proteins, glycoconjugates, amino acids, and membrane proteins are embedded in exosomes (Figure 1A). Exosomes can control cell-to-cell communication and different autocrine and paracrine phenotypes by releasing their contents to surrounding cells as paracrine signaling or faraway cells as endocrine signaling (Figure 1C) [5,14]. Endocytosis and endocytosed endocytic vesicles initiate the formation of a lipid raft region on the plasma membrane. Together with the Golgi complex, these early endosomes form late endosomes. When the limiting membrane of late endosomes invaginates into the lumen, intraluminal vesicles (ILVs) are generated. MVBs are late endosomes that store ILVs [15]. MVBs can either fuse with the plasma membrane to release their exosomes or be transferred to lysosomes for degradation [16].

According to a recent study, exosomes generated by MSCs are more effective at protecting against inflammation than microparticles released by MSCs. Multilineage differentiation of mesenchymal stem cells (MSCs) into bone, cartilage, and fat tissues distinguishes them from bone marrow and fat tissues [17,18,19,20]. The therapeutic potential of Exo-150, also known as miRNA-150-5p, generated from MSCs is also explored. Exo-150 decreases angiogenesis and synoviocyte hyperplasia, reducing joint degradation [21]. A miRNA can potentially be a biomarker to predict drug efficacy [22]. Angiogenesis, growth, differentiation, metastasis, and inflammation are all interrelated by microRNAs (miRNAs), small noncoding RNAs modulating epigenetic signals under several physiological situations. Numerous studies suggest that these molecules participate in various physiological and pathological processes. In addition to several disorders, miRNA dysregulation has been identified in RA patients. Exosomes are also vital in the pathophysiology of RA. Targeting the cargo within the recipient cells is how these biological vectors function. By targeting these charges, the behavior of recipient cells can be altered. Exosomes and their charges are important in RA patients [23]. This paper summarizes what is known regarding exosome synthesis, secretion, and immune system action mechanisms. Exosomes are important in RA disorders, which opens up a promising new way to find treatments.

## 2. Exosomes

The exosome populations are classified as EVs with a size between 40 and 100 nm [10,24]. EVs such as oncosomes, texosomes, ectosomes, microvesicles, cardiosomes, apoptotic bodies, and exosomes have been categorized according to their size, biogenesis, origin, and function [25]. Exosomes are found in body fluids like ascites, saliva, plasma, urine, cerebrospinal fluid, and breast milk [9,21]. First, endocytotic vessels are moved to early endosomes, which resemble tubes positioned at the cytoplasm’s outer edge. In the later stages of development, early endosomes convert into late endosomes, which are spherical and located closer to the nucleus. These vesicles carry a variety of information for late endosomes. During the second stage, MVBs are either destroyed by fusing with lysosomes or released as exosomes into the extracellular environment (Figure 1B) [15,26]. Exosomes are continuously produced and released by reticulocytes, mast cells, dendritic cells, B and T lymphocytes, intestinal epithelial cells, platelets, neoplastic cell lines, neurons, and microglial immune cells. Macrophages, lymphocytes, dendritic cells, and various tumor cell types produce exosomes with immunological activity [27].

According to their origin, exosomes are divided into two distinct types. Macrophages, lymphocytes, and dendritic cells are among the tumor cells that generate immunologically active exosomes. In addition to presenting antigens, they can activate, suppress, and target immune responses [15]. Exosomes and vesicles of MSC are capable of antibacterial actions as part of their immunomodulatory properties [28,29]. The exosomal content of donor cells depends on the sorting mechanism and the donor cell type. MSCs are capable of differentiating into numerous cell lineages and producing trophic factors to promote tissue repair, regeneration, and disease regression. Nonetheless, the heterogeneity of MSCs, whether innate or acquired through cultural expansion, has a substantial effect on their therapeutic efficacy. In addition, the present limitations before the purification of cell-free released peptides and exosomes include the low repeatability and uniformity of MSCs from various sources. Label-free approaches are required to better describe the MSC origin and differences across donors of these basic cultures. Developing microfluidic technologies provides new information regarding the density, shape, and size of living cells, beginning with heterogeneous or three-dimensional cultivated samples. The potential use of these technologies in the study of MSC populations may contribute to the creation of novel pharmacological methods. Hence, the capacity to identify and select an effective subset of MSCs targeting certain tissue damage or diseases is of enormous clinical importance [30,31]. Along with lipids and nucleic acids (DNA, mRNA, and small RNAs such as YRNA, miRNA, and tRNA), exosomes are composed of proteins [32]. Numerous studies have indicated that exosomes have an ever-increasing role in cell-to-cell communication [33,34]. These molecules can be released into the extracellular space or transmitted to recipient cells, revealing the biological state of the parent cells. This is important for communication between cells and tissues during homeostasis and disease, both in vivo and in vitro [5,15]. Exosomes are likely to carry ligands, receptors, antigens, and peptide-MHC complexes when immune responses are being changed. Despite being resistant to immune suppression, exosomes can propagate drug resistance via their cargo (Figure 1). The lipids in exosomes are distinct from those in their parent cells; almost nothing is known about their function [35]. EVs are also rich in sphingolipids such as ceramide, which are necessary for exosome formation. Lipids in the exosomal membrane can self-organize into “mobile rafts”, which then convert the exosomes into signalosomes and trigger cellular signaling pathways that promote tumor growth and metastasis. The signaling pathways that mobile rafts effect may be regulated, in part, by ceramide [36].

Cells that have penetrated the synovium produce exosomes into the synovial fluid. Platelet-derived exosomes are the most common type of exosomes seen in the synovial fluid of RA patients. It has also been observed that granulocytes, neutrophils, and monocytes can generate exosomes. T cells and B cells can create exosomes in the RA patient’s synovial fluid, and the number of exosomes produced by T cells correlates closely with serum levels of rheumatoid factor [5,37]. A subsequent study discovered that the creation of endosomal multivesicular bodies destroys luminal vesicles outside of the cell via the release of exosomes. There are two relevant mechanisms in this process. The first process involves the machinery of the endosomal sorting complex needed for transport (ESCRT), and the second involves tetraspanins. Exosome exocytosis remains largely unexplained. The presence of citrullinate proteins in a synovial exosome isolated from a patient with RA lends credence to the concept that exosomes play a role in the etiology of RA. Peptidylarginine deiminase, a calcium-dependent enzyme that converts arginine to citrulline after translation, produces citrulline. According to these findings, citrullination is an essential stage in the conversion of non-immunogenic proteins into auto-immune proteins, which play a crucial role in RA [38]. Johnstone et al. discovered that exosome release was linked with plasma membrane activity during reticulocyte maturation. Exosomes have a crucial role in antigen presentation and T cell activation, as discovered by Roposo et al. in 1992, and the association between exosomes and malignancies was reported in 2007. It is also reported that exosomes may transport mRNA and microRNA, suggesting that exosomes can facilitate intercellular communication by delivering nucleic acids [39]. It has been shown that exosomes can carry miRNAs to their target cells, where they control gene expression [2].

However, the levels of miRNA expression are controlled by several factors, such as isolation methodologies, sample vigor, detection procedures, and storage conditions. miRNAs are challenging to identify because of their low content, high sequence similarity, and short sequence. There are a variety of efficient and practically standard methods for identifying miRNA. As a result, miRNAs may be utilized as biomarkers for disease diagnosis, treatment response prediction, and disease progression monitoring. miRNAs can also be used to find possible biomarkers for therapeutic efficacy prediction. Developing miRNA-based treatments requires identifying the optimal miRNA targets, exploring the chemistry of miRNA, optimizing delivery, and conducting preclinical and clinical trial studies. Designing optimal delivery vehicles with reduced toxicity, increased stability, and diminished off-target effects can be a formidable obstacle [22].

## 3. Mesenchymal Stem Cell-Derived Exosomes

All cell types secrete EVs, and their function is identical to their parent cells. In rheumatic disorders, EVs isolated from synovial fluid have been shown to impact OA and RA disease progression negatively. In contrast, EVs may have a therapeutic effect by delivering chemicals that may arrest the disease’s progression [9,40]. EVs can conduct endocytosis, receptor binding, and activate intracellular signaling cascades. Additionally, they can merge with the plasma membranes of target cells [41,42]. MSCs are multipotent, ubiquitous cells that can differentiate into various mesodermal germ layer cell types [13,40,43,44,45]. MSCs have two fundamental properties: (i) they are immunomodulatory; hence, they can decrease the levels of cytokines such as interleukin 1 beta (IL-1β) and tumor necrosis factor-alpha (TNF-α) while boosting the levels of transforming growth factor beta (TGF-β); and (ii) they have a strong regeneration capacity [26]. In damaged tissue, they can divide and change into the right types of cells, but most of their regenerative effect comes from their paracrine action, which produces numerous factors [46]. MSC-derived extracellular vesicles (MSC-EVs) have the same therapeutic potential as their origin cells [26,47]. 

MSC-EVs are capable of replicating the actions of MSCs. They are comparable to MSCs in tissue regeneration, wound healing, an anti-inflammatory profile, cell migration, promoting collagen synthesis and angiogenesis, and proliferation. They are the preferred EVs for preventing the progression of disease or damage. Most of the distinctive features of MSCs are associated with their paracrine activity, particularly exosomes [19,48]. MSCs are stem cells capable of self-renewal and differentiation into skeletal and connective tissues, such as bone, cartilage, and muscle. The features of MSC-EVs are determined by the origin and growth circumstances of the cells. The principal functions of adult resident MSCs are self-repair and tissue homeostasis maintenance [47]. Advantages of using MSC-EVs over MSCs in therapy include: (i) storing and isolating ability at low temperatures; (ii) encapsulating the contents and protecting from degradation in vivo; (iii) having a longer average lifespan and being fairly stable; (iv) ability to intravenously inject and reach distant sites; (v) ability to cross the blood–brain barrier; and (vi) minimizing undesirable side effects such as immune rejection [46]. This indicates that MSC-EVs can be used as a therapeutic tool instead of the original cells, minimizing some of the adverse effects of MSCs. EVs exhibit low immunogenicity and toxicity [48]. MSC-EVs have been divided into numerous subtypes based on their origins, including human umbilical cord MSC-EVs (hUCMSC-EVs), human bone marrow-derived MSC-EVs (hBMSC-EVs), and human adipose-derived MSC-EVs (hAMSC-EVs), among others [49]. MSC-EV membranes express MSC surface markers (CD13, CD29, CD44, CD73, CD90, and CD105) but not characteristics associated with the hematopoietic system (CD34 and CD45). Additionally, the EV-specific markers CD81 and CD63 are expressed by MSC-EVs [50,51]. MSC-EVs differ from other EV types in that they contain nucleic acids such as mRNAs and miRNAs in addition to the indicators [52]. miRNAs are small non-coding RNAs consisting of 22 nucleotides [53]. Once internalized by their target cells, these miRNAs can target particular mRNAs, thereby preventing translation and potentially acting as tumor suppressors or oncogenes [10,43]. It has been shown that RNase inhibits the in vitro and in vivo effects of RNA-containing microvesicles, suggesting that these effects are RNA-dependent [54]. While EVs released by MSCs derived from human embryonic stem cells were enriched in precursor miRNAs rather than mature miRNAs, MSC-EVs primarily contain mature transcripts. Therefore, several EV types may preferentially contain distinct miRNA subtypes [43,55]. 

## 4. Exosomes as a Biomarker for RA Diagnostic

Exosomes can store data from their provenance cell, giving them potential as disease biomarkers [8,37,56]. They can leave the cells that produce them and enter the extracellular space [57,58]. The use of cell- and condition-specific cargo as biomarkers is referred to as principal exosome biogenesis [59]. External markers and protein cargo in exosomes are important diagnostic biomarkers for diseases [5,60]. As has been demonstrated, exosomes play a vital role in the physiological and pathological processes of autoimmune disorders. Consequently, extracellular exosomes can serve as diagnostic biomarkers [61]. Due to the possibility of anemia, aging, and the presence of immunoglobulins, clinically used RA biomarkers are inadequate and hinder RA diagnosis [15,62]. Therefore, identifying more efficient biomarkers is critically important [15]. Yoo et al. utilized serum exosomes from RA patients to determine the relationship between exosomal protein concentrations and disease activity. Sixty RA patients were studied using erythrocyte sedimentation, with thirty in clinical remission (CR) and thirty not in CR. Amyloid A (AA) and lymphatic vascular endothelial hyaluronic acid receptor-1 (LYVE-1) exosomal levels were not different between CR and non-CR groups, whereas LYVE-1 exosomal levels were lower in the non-CR group. Patients with higher disease activity levels had higher concentrations of exosomal AA despite having lower concentrations of exosomal LYVE-1. According to the findings, AA and LYVE-1 are useful diagnostic indicators for RA [63]. It has been determined that the lncRNA HOX transcript antisense RNA (Hotair) is a potential marker for the diagnosis of RA. Blood samples from 28 RA patients and non-RA patients with elevated C-reactive protein (CRP) levels were collected and compared. In the investigation of lncRNA in RA, BMCs and serum exosomes from RA patients showed elevated levels of HOTAIR expression. HOTAIR has a role in the pathogenesis of RA by activating MMP-2 and MMP-13 in synoviocytes of RA and osteoclasts, which may aid in the dissolution of the cartilage matrix and bone and lead to the destruction of joints. IncRNAs are more stable than mRNA transcripts and are more commonly detectable in body fluids, such as blood and urine, making HOTAIR a potentially accessible biomarker for the diagnosis of RA [64]. Biomarkers can aid in disease prognosis, diagnosis, and treatment; hence, the expansion and development of RA markers must continue. During treatment, changes in the levels of particular inflammatory mediators have been evaluated as predictors of treatment response [65,66].

## 5. The Therapeutic Ability of Exosomes in RA Treatment

For treating RA symptoms, there are several biological disease-modifying anti-rheumatic drugs (bDMARDs) with different side effects [67]. Indeed, it is vital to design novel treatment modules with fewer adverse effects. Extensive research has been conducted on the therapeutic use of exosomes of various origins [68,69]. Despite their small size, exosomes can pass through the extracellular matrix and blood vessel wall without being phagocytosed by macrophages [70]. Due to their heterogeneity, exosome proteins can enter cells in many different ways following interaction with cells [71].

Exosomes from MSCs have recently been proven to promote cartilage regeneration when utilized as carriers for therapeutic compounds [72]. By using exosomes, many of the risks of cell-based treatments, such as transplanted cells turning into cancer, immune system rejection, and ossification and calcification, can be reduced [73]. Exosomes regulate immunological reactivity, stimulate proliferation, and inhibit apoptosis to facilitate cartilage regeneration. MSC exosomes have initiated and promoted chondrocyte migration and proliferation, and concurrently, chondrocytes inhibit apoptosis. The production of the chondral matrix is accelerated by stimulating the AKT and/or ERK signaling pathways. In addition to the beneficial effect of MSC-derived exosomes, it is impossible to omit a crucial factor. At the lesion region, there are fewer proinflammatory synovial cytokines (e.g., TNF-α and IL-1β) and more CD163^+^ regenerative M2 macrophages [74]. The selected studies on exosome-based treatment for RA according to source, sub-type factor, study model, and biological effects are summarized in Table 1.

MSCs-derived exosomes are an appropriate vector for the transfer of therapeutic miRNA-124a. If this cell was co-incubated with the stated exosome, it induced apoptosis and prevented the migration and proliferation of the FLS cell line. Exosomes are produced from human MSCs that overexpress miRNA-124a. Upon co-incubation with HMSC-124a-EV, these exosomes inhibit the migration and proliferation of FLS cells and enhance their apoptosis [81]. Rheumatoid arthritis fibroblast-like synoviocytes (RA-FLSs) can endocytose exosomes of MSCs with high miR-320a levels. In RA-FLSs, endocytic exosomes release miR-320a, and miR-320a can inhibit CXCL9. CXCL9 promotes RA-FLS invasion and migration by increasing the expression of IL-1, IL-6, and IL-8. A high level of miR-320a expression reduces CXCL9, inhibiting RA-FLS invasion, activation, and migration, and in the CIA model, mice suppress the production of RA and immune factors [80]. According to the analysis of RA patients, angiogenesis and expression of VEGF and MMP14 were raised, whereas expression of miR-150-5p was diminished. It has been discovered that miR-150-5p can influence angiogenesis. It was discovered that miR-150-5p targets and adversely controls VEGF and MMP14 in FLS in a direct manner. Exo-150 suppresses angiogenesis and prevents RA FLS invasion and migration by negatively regulating VEGF and MMP-14. MMPs are associated with synovial inflammation, which destroys articular cartilage. Targeting MMPs could be a potential treatment option for inflammatory joint disorders [79]. Exosomes from stem cell-derived mesenchymal stromal cells are excellent for transferring therapeutic miRNA.

**Figure 2:**
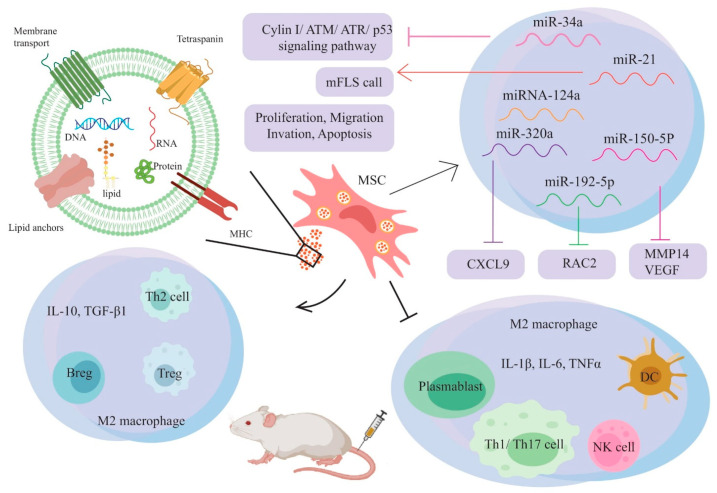
Experimental treatment and immunomodulatory effects of MSC-EVs in RA.

Exosomes derived from human umbilical cord mesenchymal stem cells (hUCMSC-exos) were examined for their beneficial effects on bone destruction in CIA rats. hUCMSC-exos was able to treat bone degeneration in CIA rats without causing any adverse side effects. Therefore, the therapy prevented joint synovial hyperplasia and inhibited the migration of inflammatory cells to the joints. According to their findings, RANKL concentrations decreased in the serum and synovial tissues of CIA rats, whereas OPG concentrations increased. It postulated that an imbalance between RANKL and OPG could be the underlying mechanism for inhibiting bone destruction [86]. MSCs may regulate T cell differentiation and proliferation as well as inhibit proinflammatory factor production. The performance of AT-MSCs derived from human adipose tissue influenced the generation of T regulatory cells (Tregs). They create IL-10 and reduce the immune response, but they inhibit the development of activated CD4^+^ T cells into T helper (T_H_) 17 cells, which produce interleukin (IL)-17 [87]. When the amount of HAND2-AS1 was enhanced in RA FLSs by co-culturing them with hMSCHAND2-AS1-Exos, the expression of miR-143-3p was inhibited, and the expression of HAND2-AS1 was elevated. HAND2-AS1 was found to increase the expression of TNFAIP3, which is a direct target of miR-143-3p. In RA-FLSs, HAND2-AS1 was discovered to decrease the amount of p-p65, one of the transcription factor families of NF-κB. Consequently, HAND2-AS1 overexpression in exosomes derived from MSCs inhibited progression and induced apoptosis in RA-FLSs by inactivating the NF-κB pathway via the TNFAIP3/miR-143-3p axis [88]. Recent advances in exosome research will disclose the functional pattern and precise properties of exosomes in autoimmune diseases such as RA [89,90]. Exosome-based therapy could provide a safe and new method of treating arthritis. Figure 2 shows in detail how MSC-EVs were used in an experiment for RA treatment and how they affected the immune system. Overall, using exosomes to treat arthritis can be regarded as a unique, safe, and adequate therapeutic approach.

## 6. Conclusions

This paper focuses on the therapeutic potential of exosomes. Exosomes convey various molecules, including lipids, RNA, DNA, proteins, and microRNA. Exosomes derived from MSCs have demonstrated the same therapeutic benefits in autoimmune disorders as their origin cells. In addition, mesenchymal-derived exosomes possess immunomodulatory capabilities; however, tailoring these vesicles with anti-inflammatory molecules and specific receptors may enable them to target specific tissues/organs. miRNAs play an important role in determining the therapeutic efficacy of MSC-exosomes in various disorders, and MSC-exosomes are the subject of an expanding body of research. In recent years, a few studies have been undertaken in this field; however, certain challenges remain. Natural carriers, such as exosomes, have advantages and disadvantages compared to synthetic carriers (liposomes or nanoparticles). This includes reduced levels of toxic and immunogenic features, greater stability, longer-lasting maintenance, a low risk of aneuploidy, and a low risk of immunological rejection following in vivo allogeneic injection. The therapeutic potential of exosomes cannot be denied. In addition, several of them can be regarded as valid biomarkers for detecting or even diagnosing RA disease activity. RA, a disease that has resulted in considerable impairment in individuals, has a global influence on people’s lives. Several risk variables, including age, smoking, hormones, lifestyle changes, genetics, and epigenetics, are implicated in the pathophysiology of this inflammatory joint disease. Multiple studies have demonstrated the involvement of distinct molecular and cellular signaling pathways in the initiation and progression of RA. A considerable improvement in targeted drug delivery and decreased side effects has been obtained using nanoparticulate technologies. In the future, nanotechnology can potentially improve disease diagnosis, treatment, and research.

## Figures and Tables

**Table 1:** Overview of studies on exosome-based RA treatment.

Source	Sub-Type Factor	Study Model	Biological Effects	Ref.
hUCMSCs	sEVs	CIA rats	Decreasing: immunomodulatory effect of T lymphocytes Synovial hyperplasia, synovial inflammatory cell infiltration, paw oedema and arthritis index	[75]
AMSCs	MVs	IL-1Ra−/− mouse BALB/c mouse	Decreasing: IL-1Ra contained in EVs transferred IL-1β, IFN-γ, and TNF-α in serum, synovial hyperplasia and pannus formation, thickness of joints of paws	[76]
hUCMSCs	EVs	normal group rats CIA rat model group	Restoring the balance of Treg/Th17 inside the spleen, regulating the expression of RORγt and Foxp3 inside joints, inhibiting IL-17, and enhancing the expression of TGF-β in the serum, consequently improves arthritis	[77]
BMSCs	miR-21	BALB/c mice CIA mice	Promoting mFLS cell proliferation and suppressing inflammatory cytokine secretion by targeting the TET1/KLF4 regulatory axis, thereby miR-21 reduces RA progression	[78]
BMSC Wistar rats, bone marrow Mouse bone marrow-derived mesenchymal stem cells	miR-192-5p	CIA rat	Reduction of RAC2 expression in synovial tissues. Blocking the inflammatory response by reducing levels of the pro-inflammatory cytokines PGE2, IL-1, TNF-, NO, and iNOS	[18]
BMSC DBA/1J mice, bone marrow	miR150-5p	CIA rat	Inhibition of Pro-inflammatory Cytokine-Induced Invasion, Migration, and Tube formation by Targeting and downregulating MMP14, VEGF in FLS. modulate angiogenesis	[79]
BMSC RA patients, bone marrow	miR-320a	CIA mouse	Inhibition of FLS activation, migration, and invasion in RA by targeting CXCL9 expression suppression results in attenuation of arthritis and bone damage.	[80]
Human MSCs	miRNA-124a	NR	After co-culture of MSC exosome miR-124a with RA-FLS, it arrested the MH7A cell cycle in G0/G1 phase, reducing wound closure percentage and wound healing rate; Proliferation, invasion, migration, and promoted apoptosis RA-FLS inhibited	[81]
BMSCs	EVs MPs Exosomes	DTH Mouse CIA Mouse	The immune-modulating effect in the inflammatory arthritis model by reducing the proliferation of T and B lymphocytes and inducing the Treg cell population, increasing Treg cells, and reducing plasmablast differentiation, thus reducing bone destruction and erosion	[82]
BMSCs	SEVs miR-34a	RA rat	Reducing RA inflammation by inhibiting the cyclin I/ATM/ATR/p53 signaling pathway; inhibition of RA-FLS proliferation and resistance to apoptosis in vitro	[83]
Human neutrophils	MVs	K/BxN arthritis mouse model	neutrophil-derived AnxA1+ MVs reduced cartilage destruction decreasing	[84]
G-MDSCs	sEVs	CIA mouse	The proportion of IL-10 producing B cells by prostaglandinE2 exosomal stimulation c, plasma cells, and follicular helper T cells as well as CIA development in mice decreasing	[85]

## Data Availability

Not applicable.
